# Supplementary material for: Optimization of differentiation and transcriptomic profile of THP-1 cells into macrophage by PMA
Source: PLoS One. 2023 Jul 17;18(7):e0286056. doi: 10.1371/journal.pone.0286056 (PMC10351730; doi:10.1371/journal.pone.0286056)
Supplement: S1 File — (ZIP) [file pone.0286056.s001.zip › supporting files/RNA-seq report.zip!/RNA-seq report/4_GOEnrichment_result/induce_vs_control/DAG/induce_vs_control.DEG_mf_DAG.pdf]

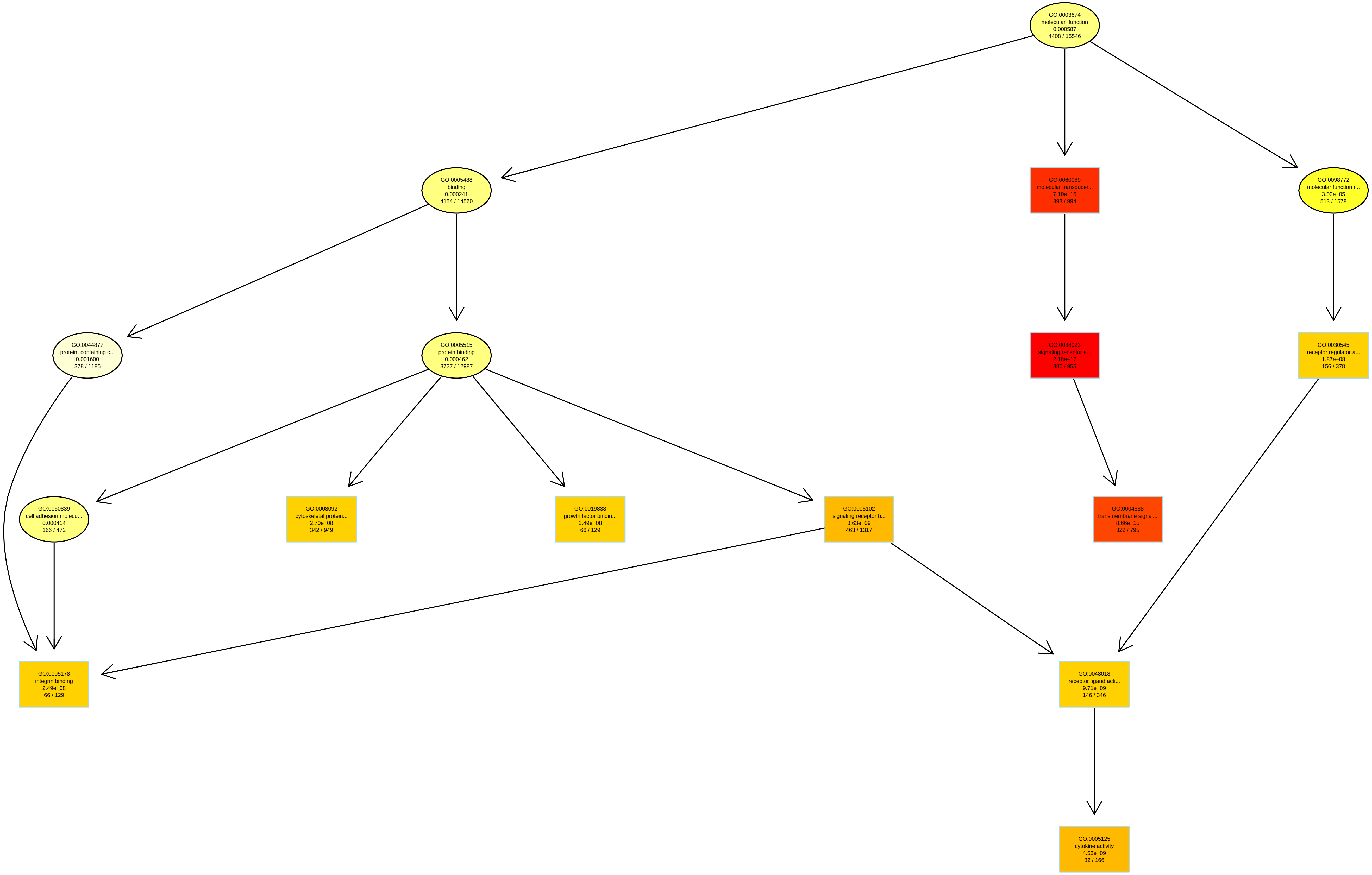

GO:0003674
molecular_function
0.000587
4408 / 15546
GO:0005488
binding
0.000241
4154 / 14560
GO:0060089
molecular transducer...
7.10e−16
393 / 994
GO:0098772
molecular function r...
3.02e−05
513 / 1578
GO:0044877
protein−containing c...
0.001600
378 / 1185
GO:0005515
protein binding
0.000462
3727 / 12987
GO:0038023
signaling receptor a...
2.18e−17
386 / 955
GO:0030545
receptor regulator a...
1.87e−08
156 / 378
GO:0050839
cell adhesion molecu...
0.000414
166 / 472
GO:0008092
cytoskeletal protein...
2.70e−08
342 / 949
GO:0019838
growth factor bindin...
2.49e−08
66 / 129
GO:0005102
signaling receptor b...
3.63e−09
463 / 1317
GO:0004888
transmembrane signal...
8.66e−15
322 / 795
GO:0005178
integrin binding
2.49e−08
66 / 129
GO:0048018
receptor ligand acti...
9.71e−09
146 / 346
GO:0005125
cytokine activity
4.53e−09
82 / 166
